# Neuroprotective Potential of GDF11: Myth or Reality?

**DOI:** 10.3390/ijms20143563

**Published:** 2019-07-21

**Authors:** Luc Rochette, Gabriel Malka

**Affiliations:** 1 Equipe d’Accueil (EA 7460), Physiopathologie et Epidémiologie Cérébro-Cardiovasculaires (PEC2), Université de Bourgogne—Franche Comté, Faculté des Sciences de Santé, 7 Bd Jeanne d’Arc, 21000 Dijon, France; 2 Institut de Formation en Biotechnologie et Ingénierie Biomédicale (IFR2B), Université Mohammed VI Polytechnique, 43 150 Ben-Guerir, Morocco

**Keywords:** neuroprotection, rejuvenating factors, GDF11

## Abstract

In the brain, aging is accompanied by cellular and functional deficiencies that promote vulnerability to neurodegenerative disorders. In blood plasma from young and old animals, various factors such as growth differentiation factor 11 (GDF11), whose levels are elevated in young animals, have been identified. The blood concentrations of these factors appear to be inversely correlated with the age-related decline of neurogenesis. The identification of GDF11 as a “rejuvenating factor” opens up perspectives for the treatment of neurodegenerative diseases. As a pro-neurogenic and pro-angiogenic agent, GDF11 may constitute a basis for novel therapeutic strategies.

Neurodegenerative diseases are situations that induce the progressive loss of neuronal integrity accompanied by cellular and functional deficiencies. In the United States, 46 million people are over the age of 65, and the number of older adults is predicted to increase to 24% of the population, about 98 million people, by 2060. In the United Sates, Alzheimer’s disease, primarily seen in adults over the age of 65, affected 5 million people in 2013 [1].

Aging is characterized by an increasing homeostatic imbalance, accompanied by a gradual decline in the regenerative properties of the cells. Proliferation and differentiation of neural stem/progenitor cells residing within the healthy brain are essential in inducing these processes. New results suggest aging brains can potentially be rejuvenated. Recent studies indicate that adult neurogenesis may produce not only young and excitable new neurons but also completely new neuronal subtypes [2,3]. Identifying the molecular mechanisms of degenerative changes, regeneration, and rejuvenation is important for developing therapies to treat age-related diseases. 

The brain is more vulnerable to oxidative stress than other organs. Although the brain only accounts for ~2% of body mass, it utilizes 15–20% of the energy generated in the whole body. Fundamentally, it is proposed that aging is the consequence of the progressive generation of reactive oxygen species (ROS) that damage macromolecules such as DNA and cause neurodegenerative disorders [4].

The brain must rely on the circulation for the continuous supply of nutrients and oxygen and for the disposal of metabolic waste products [5]. Conventionally, it has been considered questionable whether the nervous tissue can regenerate, because mature neural cells are very limited in their ability to proliferate or differentiate. In the adult mammalian brain, three areas are neurogenic and contain neural stem cells: The subgranular zone in the hippocampal dentate gyrus, the subventricular zone nearby the lateral ventricles, and the hypothalamus. Neural stem cell niches have been identified, regulating neural stem cell activity [6]. Stem cells such as adipose-derived stem cells (ADSCs) could transdifferentiate into neuron-like cells and present neuronal properties [7]. There is evidence that exercise has favorable effects on the number and function of different circulating angiogenic cells. The molecular changes evoked by exercise may contribute to a more powerful ADSC action in the brain. Exercise has been shown to improve a wide range of age-related cellular and functional impairments [8].

Many molecular mechanisms in liaison with excess of ROS in the brain may contribute to human aging. Cellular senescence, a state associated with distinct changes of specific gene expression and the acquisition of a complex proinflammatory secretory profile, has emerged as a potentially important contributor to aging and age-related diseases [9]. Potential triggers of cellular senescence and neurogenesis were extensively studied, especially in the context of functional impairment of the remaining stem cells [10]. It is widely known that, in the cerebral structures, the control of the neurovascular unit depends on the regulated levels of ROS. Gradually, physiological levels of ROS exceed the brain’s innate defenses, and then the neuronal functions decline. The degeneration involves the neurovascular inflammatory process, neurons, neuroglia, and blood vessels. The “neurovascular unit” concept challenges the symbiotic relationship between brain cells and cerebral blood vessels. The causes of several neurological disorders may implicate various factors, including the onsets of cerebrovascular inflammation [11]. The age-associated neurodegenerative disease Alzheimer’s disease is characterized by the accumulation of aggregated amyloid-beta (Aβ) peptides in the brain. A relationship between Aβ aggregation and cellular stimulation of proinflammatory cytokine release has been demonstrated These amyloid peptides induce changes in the microglial phenotype and activate signaling cascades such as the NLRP3 inflammasome [12]. 

Astrocytes, microglia, and immunocytes play vital roles in the brain and are involved in neuro-inflammatory diseases. The infiltration of leukocytes from blood vessels into the central nervous system (CNS) has been implicated in the pathogenesis of neurological disorders associated with inflammation. At the start of an inflammatory response, pro-inflammatory mediators induce changes in the endothelial coating of the blood vessels and in leukocytes. This process results in amplified vascular permeability and expression of adhesion proteins, promoting the adhesion of neutrophils to the endothelium with consequent neurodegeneration [13]. Various mechanisms influence neurovascular inflammation and neurodegeneration, and in this field, many studies have described that autophagy plays a major role in the process of neurodegeneration and in the limitation of regeneration [14]. Limitation of neurovascular injury is a promising approach to control neuroinflammation. 

Aging is characterized by the reduced regenerative capacity of tissues. Tissue regeneration and rejuvenation in aging organisms has been studied after heterochronic parabiosis (HP) [15]. In order to investigate the influence of an organism on its conjoined partner, animal models that “mimic” the natural phenomenon of conjoined twins were created. The surgical technique of physically connecting two living organisms is termed “parabiosis”, from the Greek “para” (next to) and “bios” (life). Collecting verification has indicated that the blood of young animals holds powerful “factors of youth”. Rejuvenating effects of HP are observed in the aged brain, indicating that young blood counteracts cellular and functional age-related neuronal decline [16]. The endogenous compound growth differentiation factor 11 (GDF11), present in young blood, is a circulating negative regulator of cardiovascular and neuronal functions, suggesting that raising GDF11 levels could potentially treat or prevent various age-related diseases [17].

GDF11 is produced from precursor proteins by proteolytic processing. There is a high degree of identity between the active domains of mature growth factors such as GDF8/myostatin and GDF11. GDF11 has been measured in different tissues. Recently, using immunohistochemistry, GDF11 expression in almost all neurons throughout the rat brain and spinal cord was demonstrated. To investigate the developmental changes in GDF11 expression, immunohistochemistry was performed using rat brains at different ages. The expression levels peaked at two postnatal weeks and then gradually decreased [18]. The broad GDF11 distribution suggests that it may have critical functions for neurons. GDF11 is highly expressed in many embryonic murine tissues, including the developing CNS. It has been reported that GDF11 facilitates the temporal progression of neurogenesis in the developing spinal cord [19]. Its expression is most abundant in young adult organs and seems to decrease during aging [20]. Today, it is still controversial whether the tissue levels of GDF11 protein are age-related. The hippocampus has been studied extensively for age-related structural and functional impairments as well as age-dependent deficits in cognition. A wide variation of GDF11 protein expression was detected in the hippocampus of old animals. Moreover, interestingly, GDF11 appeared to be differently modulated by exercise in the hippocampus of young and old mice [21]. A recent study established that GDF11 enhanced hippocampal neurogenesis and plasticity in the hippocampus and cortex of old mice [22].

Studies have focused on GDF11 as a potential anti-aging regulating molecule. Evidence indicates that it may function as a pro-neurogenic and pro-angiogenic agent (Table 1). Consistent with these properties, GDF11 treatment may protect the brain, and its administration may be a viable approach for improving deleterious aspects of brain aging and neurodegenerative diseases. Recombinant GDF11 (rGDF11) treatment improves the cerebral vasculature and enhances neurogenesis [23,24]. In the context of aging, GDF11 appears to promote vascular and neural plasticity of the central nervous system [22,25,26]. It is suggested that cognitive improvements elicited by young blood administrated to animals may not be limited to neurogenesis but may also be due to enhancements in synaptic plasticity [27]. Biochemical experiments have demonstrated that GDF11 can activate Smad2, suggesting the involvement of ALK receptors in GDF11 signaling [25] (Figure 1). Investigation of GDF11 in the cerebrospinal fluid (CSF) has been studied. In patients with amyotrophic lateral sclerosis (ALS), higher CSF levels of GDF11 were correlated with a better disease issue [28]. In this complex field, GDF11 biology suggests that it is a major regulator of brain functions in connection with other growth and trophic factors. Neuronal temporal identity is multifactorial. Spatial and temporal growth factors influence each specialized cell type. Brain anatomy includes multiple specialized cells that function together through the neurovascular unit. This unit appears fundamental for brain metabolism and is regulated by numerous endogenous modulators. In this field, inhibition of inflammation appears to be a potential therapeutic strategy for neuro-inflammatory injury, and the role of GDF11 may be evoked [29].

A number of experimental and clinical studies investigated the role of GDF11 on stroke recovery. In the model of experimental stroke induced by cerebral ischemia/reperfusion, neuro-inflammatory injury is present. Systemic administration of rGDF11 exerted proangiogenic effects following cerebral ischemia and contributed to improve the neurological function in this model of stroke. After ischemic injury, GDF11-induced angiogenesis was probably mediated by TGF-β signaling [25]. In stroke patients, angiogenesis is an important factor for stroke recovery. Consistent with its pro-neurogenic and pro-angiogenic properties in the adult brain, GDF11 treatment may improve stroke condition [31]. The beneficial effects of GDF11 on neurogenesis in the aged brain may be closely dependent on the improvement of the vascular function, as GDF11 increases the concentration of vascular endothelial growth factor (VEGF), a potent angiogenic factor.

The pathogenesis of neurodegenerative diseases is unclear. It is stated that Alzheimer’s disease is a complicated heterogeneous disease involving multiple factors, including heredity factors, neurotransmitters, immune and environmental factors. The regenerative abilities of a cell depend on the maintenance of a functional proteome. High profile studies have reported conflicting data regarding the properties of GDF11. Increasing GDF11 levels may improve some facets of Alzheimer’s pathology [26,30]. A large number of studies have found confirmation that the key characters of Alzheimer’s disease are changes in synaptic plasticity and neuron injury. Neurons are cells highly dependent on mitochondrial oxidative phosphorylation for energy production. GDF11 reduces oxidative stress and mitochondria damage. After exogenous injection of rGDF11, apoptosis and inflammation are reduced [32]. Oxidative stress and inflammation are proved to have essential roles in cerebral injuries. Heme-oxygenase-1 (HO-1), a major antioxidant, protects cells and tissues against the attack of oxidative products in the presence of brain injuries [33]. It is proposed that the role of GDF11 in suppressing ROS overproduction contributes to the limitation of inflammation and of the level of mitochondrial injury. Many pro-longevity signaling pathways, such as forkhead box class O (Foxo) transcription factors and SIRT1, have been shown to play important roles in brain function. The manipulation of signaling molecules that impact Foxo and SIRT1 activities improves the neuronal stress response associated with ROS production. 

As we reported, several factors that are elevated in young animals were identified in blood plasma from mice, and their blood concentrations inversely correlated with the age-related decline in neurogenesis. In contrast to GDF11, the levels of a new factor, CCL11, increase with age in animals. CCL11 is a C–C motif chemokine 1 [34]. CCL11 injected into young animals led to degenerative changes in the CNS, disturbed the cognitive functions, and inhibited tissue regeneration. The identity of specific factors in the blood of young mice mediating cognitive functions and their potential molecular pathways have not been clearly elucidated yet.

In conclusion, among the TGFβ proteins that regulate aspects of CNS formation and health, GDF11 is a ‘‘rejuvenating factor”, able to treat diseases in different systems in old individuals and a factor that promotes neurogenesis in aged mice. Recent studies have shown that stimulating the intrinsic growth potential of CNS neurons can induce axon regeneration after CNS injury. The identification of GDF11 as a “rejuvenating factor” opens up perspectives for the treatment of age-related brain dysfunction.

## Figures and Tables

**Figure 1:**
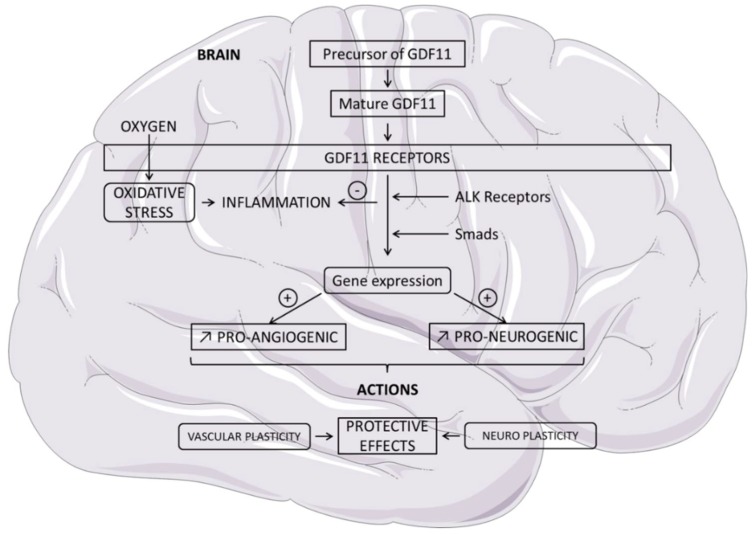
Schematic overview of the pro-angiogenic and pro-neurogenic properties of growth differentiation factor 11 (GDF11) in the brain. Mature GDF11 exerts its effects by binding to activin receptor-like kinases (ALK) inducing the phosphorylation of Smads. GDF11 reduces oxidative stress and inflammatory process.

**Table 1:** Pro-angiogenic and pro-neurogenic properties of GDF11 in aging and disease models.

Treatments GDF11	Model Type	Effects	References
Recombinant GDF11 (rGDF11) Intraperitoneal (i.p.) 0.1 mg/kg Daily injection for 28 days	Old mice (23-month-old)	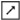 angiogenesis 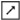 neurogenesis	[24]
rGDF11 1 mg/kg i.p. Daily injection for 28 days	Young (2–3-month-old) and old mice (22–23-month-old)	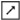 angiogenesis neuronal activation	[22]
rGDF11 0.1 mg/kg i.p. 1 day	Young mice (1–5-month-old) and mice (9-month-old)	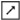 neurogenesis 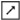 short term memory	[30]
rGDF11 0.1 mg/kg i.p. For 7–13 days after stroke	Mouse model of stroke (right middle cerebral occlusion: RMCO)	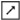 angiogenesis 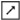 neurogenesis	[31]
rGDF11 0.1, 0.2, 0.3 mg/kg i.p. For 7 days	Rat model of stroke (RMCO and reperfusion)	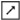 angiogenesis 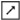 endothelial proliferation	[25]
rGDF11 Intravenous 0.1 mg/kg For 28 days	Mouse model of Alzheimer’s disease	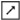 angiogenesis 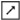 neurogenesis	[26]
